# Impact of enhanced recovery after surgery protocols on patient-reported outcomes and satisfaction following shoulder arthroplasty: a systematic review

**DOI:** 10.1016/j.xrrt.2026.100725

**Published:** 2026-03-19

**Authors:** Hussayn Shinwari, Asmaar Butt, Hanan Taimur Shinwari, Zakariya Mouyer, Saran Singh Gill, Abith Ganesh Kamath, Kapil Sugand

**Affiliations:** a Faculty of Medicine, St George's University of London, London, UK; c Faculty of Medicine, Imperial College London, London, UK; b Faculty of Medicine, University of Sheffield, Sheffield, UK

**Keywords:** Shoulder arthroplasty, Enhanced recovery after surgery, Postoperative rehabilitation, Analgesia protocols, Patient-reported outcomes, Opioid-sparing, Interscalene nerve block, Functional recovery

## Abstract

**Background:**

As shoulder arthroplasty volume rises globally, evidence on optimal perioperative rehabilitation and analgesia remains limited. This systematic review evaluated enhanced recovery after surgery postoperative strategies on patient-reported outcomes and satisfaction.

**Methods:**

PubMed, Embase, MEDLINE, Global Health, and Cochrane Library were searched per Preferred Reporting Items for Systematic Reviews and Meta-Analyses 2020. Studies undergoing total shoulder arthroplasty or reverse total shoulder arthroplasty (RTSA) were included. Interventions: rehabilitation timing and analgesic protocols. Outcomes: American Shoulder and Elbow Surgeons (ASES) score, Simple Shoulder Test (SST), Single Assessment Numeric Evaluation (SANE), range of motion, Quality of Recovery-15, and satisfaction.

**Results:**

Eighteen studies including 1,722 patients (total shoulder arthroplasty: n = 7; RTSA: n = 2; mixed: n = 9) were analyzed. Delayed rehabilitation after RTSA improved early pain scores (ASES pain 26.3 ± 16.3 vs. 16.7 ± 11.6 at 6 months; *P* = .01) and overall function (ASES total 40.2 ± 20.1 vs. 30.0 ± 18.8; *P* = .04), without affecting long-term outcomes (ASES 88-90, SST 9.8-9.9, SANE 85-88 at 12 months). Opioid-sparing and opioid-free protocols achieved equivalent functional recovery (ASES 74-89, SST 6-9, SANE 24-28 change-from-baseline) and consistently higher patient satisfaction (86-97%) at 2-6 weeks. Continuous interscalene blocks and liposomal bupivacaine provided effective analgesia (Quality of Recovery-15 postoperative day 3: 124.5-132.0), with trends toward improved early recovery. Across studies, range of motion outcomes (forward flexion 126-146° and external rotation 57-62°) were comparable, indicating multiple enhanced recovery after surgery strategies can deliver similar functional and patient-reported outcomes while enhancing early recovery and satisfaction.

**Conclusion:**

Functional recovery following shoulder arthroplasty appeared robust across diverse rehabilitation and analgesic strategies. Delayed rehabilitation offered early pain advantages, and opioid-sparing approaches enhanced patient satisfaction without compromising outcomes. These findings supported a personalized, patient-centered approach to postoperative care and highlight the need for further high-quality comparative trials.

Enhanced recovery after surgery (ERAS) is a comprehensive, multimodal approach to perioperative care designed to minimize the physiological stress of surgery and facilitate faster recovery.^6^^,^^38^ Grounded in evidence-based practice, ERAS protocols encourage the standardization and continuous optimization of perioperative interventions to improve clinical outcomes while reducing complications, length of stay, and overall health-care expenditure.^36^^,^^58^ ERAS does not refer to a single intervention but represents an integrated, evidence-based framework that combines preoperative education, optimized analgesia, opioid-sparing (OS) strategies, early or criteria-based mobilization, and targeted nutritional and fluid management.^15^^,^^22^^,^^32^^,^^37^^,^^41^^,^^43^^,^^49^, ^50^, ^51^^,^^59^^,^^62^^,^^65^^,^^67^^,^^68^^,^^75^^,^^76^ Its goal is to standardize perioperative care while enhancing recovery and patient outcomes across multiple domains.

Patient-reported outcome measures (PROMs), defined as standardized and validated tools used to capture patients' own perceptions of their health, function, and quality of life, are increasingly recognized as essential endpoints in surgical evaluation.^55^ Their use in lower-limb arthroplasty has demonstrated that ERAS protocols can enhance recovery without compromising patient satisfaction or quality of life.^29^ Moreover, PROMs provide clinicians and patients with meaningful insights into treatment efficacy and functional outcomes, offering a more patient-centered perspective than traditional clinical or radiographic assessments.^1^^,^^3^^,^^8^^,^^9^^,^^16^^,^^18^^,^^19^^,^^21^^,^^24^, ^25^, ^26^^,^^30^ This is particularly important in shoulder arthroplasty (SA), where patient satisfaction is often driven by nuanced goals such as the ability to perform specific tasks, pain relief, or a sense of joint stability that may not correlate with radiographic or objective and measurable clinical outcomes.^70^^,^^73^

Although several reviews have examined individual ERAS components, such as perioperative analgesia with liposomal bupivacaine (LB) or interscalene blocks, there is no synthesis evaluating the combined effects of analgesic strategies and rehabilitation protocols on patient-reported outcomes and satisfaction following SA.^31^^,^^72^ Existing studies often focus on isolated interventions, limiting guidance for holistic, patient-centered ERAS pathways.

## Aim

This systematic review therefore aimed to evaluate how ERAS components specifically postoperative rehabilitation and analgesic strategies affect patient-reported outcomes and satisfaction after SA.

### Methodology

#### Adherence to guidelines

**Figure 1:**
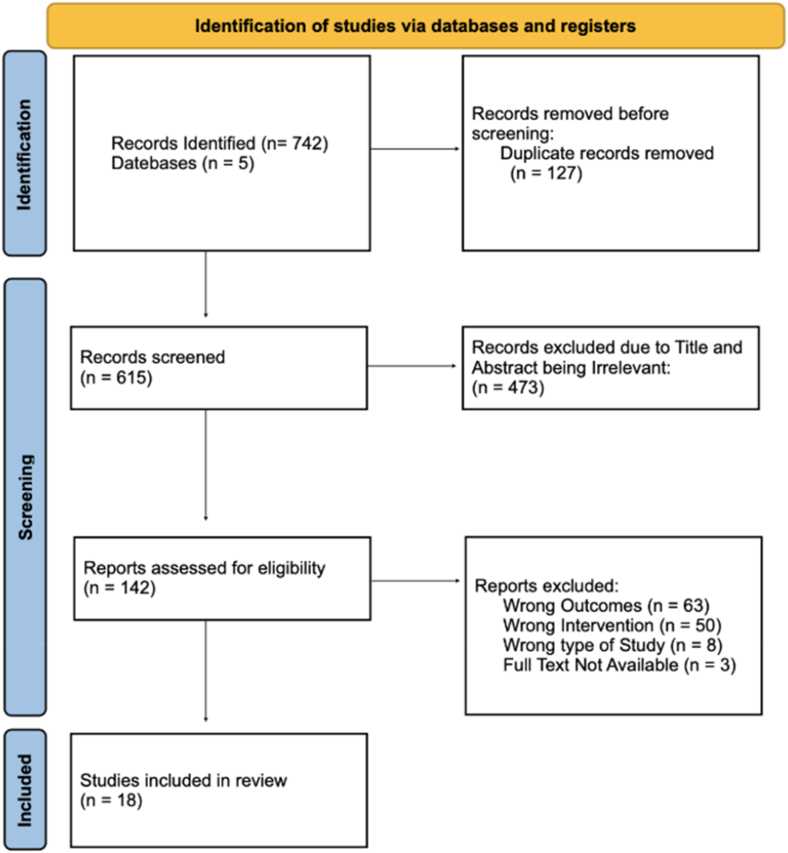
PRISMA flow diagram of study selection.

This review was carried out in accordance with the Preferred Reporting Items for Systematic Reviews and Meta-Analyses 2020 guidelines.^44^ A visual summary of the study selection process is presented in Fig. 1. The approach to evidence synthesis followed the Synthesis Without Meta-analysis recommendations.^4^ The review protocol was prospectively registered on the International Prospective Register of Systematic Reviews (PROSPERO) under the registration number CRD420251036859.

#### Database search strategy

A comprehensive search was conducted across multiple databases, covering literature from inception up to February 14, 2025. Databases searched included PubMed, the Cochrane Library, and, through the OVID platform, Medline, Embase, and Global Health. The complete search strategy is detailed in Supplementary Table 1.

#### Eligibility criteria

Eligible studies included adults (≥18 years) undergoing primary or revision total shoulder arthroplasty (TSA), reverse total shoulder arthroplasty (RTSA), or hemiarthroplasty for all indications, including osteoarthritis, rotator cuff arthropathy, inflammatory arthritis, and proximal humeral fractures. Studies were required to implement at least 1 ERAS component, specifically the following: (i) postoperative rehabilitation (eg, early versus delayed mobilization, passive versus active-assisted exercises), (ii) analgesic strategies (eg, multimodal analgesia, OS or opioid-free regimens, interscalene nerve blocks, LB, local infiltration analgesia), or (iii) perioperative optimization measures (eg, preoperative education, tranexamic acid (TXA) administration, sleep pathway interventions). Included studies had to report PROMs such as the American Shoulder and Elbow Surgeons (ASES) score, Simple Shoulder Test (SST), Single Assessment Numeric Evaluation (SANE) score, Quality of Recovery-15 (QoR-15), range of motion (ROM), or patient satisfaction, and include a comparator group. Exclusion criteria were nonshoulder procedures, pediatric populations (<18 years), or studies lacking ERAS components, relevant outcomes, or a control arm. Detailed inclusion and exclusion parameters are provided in Supplementary Tables 2 and 3. The Population, Intervention, Comparator, Outcomes framework guided study selection and data extraction.

#### Screening and data extraction

References were imported into Covidence, where duplicate entries were removed. Two reviewers (H.S. and A.B.) independently screened titles and abstracts, adopting a deliberately inclusive approach in accordance with Cochrane Handbook guidance to avoid premature exclusion. Full-text screening of shortlisted studies was then undertaken using predetermined Population, Intervention, Comparator, Outcomes–based criteria.^10^^,^^53^ Data extraction was carried out independently by 4 reviewers (H.S., Z.M., A.B., and H.T.S.). Discrepancies arising during screening or extraction were addressed through consensus, with unresolved conflicts arbitrated by the senior author (K.S.). Key information extracted included study title, authorship, country of origin, publication year, evaluated outcomes, study conclusions, and sample sizes. Data were logged in Microsoft Excel (Microsoft, Washington, USA). Where required information was missing, authors were contacted for clarification.

#### Risk of bias

**Figure 2:**
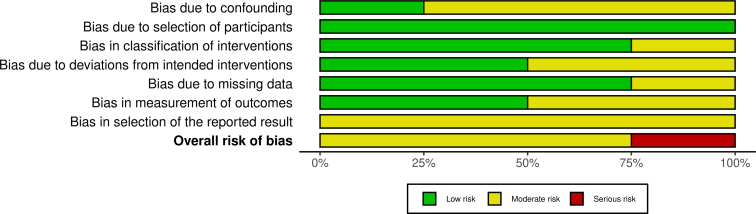
Risk of bias assessment of non-randomised studies (ROBINS-I tool).

**Figure 3:**
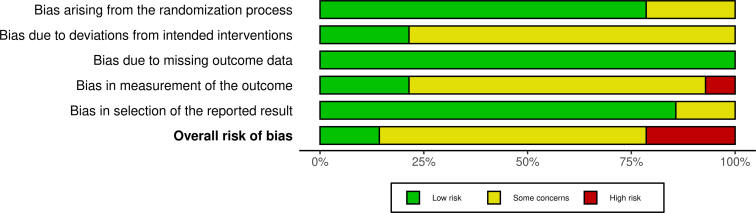
Risk of bias assessment of randomised controlled trials (RoB 2 tool).

Risk of bias was evaluated independently by 2 reviewers (Z.M. and A.B.). For randomized controlled trials (RCTs), the revised Cochrane risk-of-bias tool (RoB 2) was employed, while the ROBINS-I tool was used for nonrandomized studies.^60^^,^^61^ Figure 2, Figure 3 presented the outcomes of these assessments. Any disagreements were resolved through discussion, with HS acting as the final adjudicator when needed. Domains assessed included randomization procedures, blinding, and the management of missing outcome data.

#### Data analysis, qualitative synthesis, and reporting

Given the variability in study designs, surgical techniques, and outcome measures, statistical pooling of results via meta-analysis was not appropriate. A narrative synthesis was therefore performed following Synthesis Without Meta-analysis guidance, providing a structured summary of findings across studies.^4^

## Results

### Study selection

A total of 742 articles were initially identified through comprehensive database searches of PubMed/MedLine, Cochrane Library, Embase, and Global Health (Fig. 1). After removal of duplicates and detailed screening against predefined inclusion and exclusion criteria (Supplementary Tables 2 and 3), 18 studies involving a total of 1,722 patients were deemed eligible and included in the final analysis^5^^,^^7^^,^^11^^,^^14^^,^^17^^,^^23^^,^^27^^,^^28^^,^^34^^,^^39^^,^^45^, ^46^, ^47^, ^48^^,^^52^^,^^56^^,^^57^^,^^63^ (Table I).

**Table I:** Study characteristics.

Authors (yr)	Title	Journal	Aims/methodology	Conclusion	Country	Study design	Sample size	Male (%)	Age in yr (mean ± SD)	BMI (mean ± SD)
Hagen et al^24^ (2020)	A randomized single-blinded trial of early rehabilitation versus immobilization after reverse total shoulder arthroplasty	Journal of Shoulder and Elbow Surgery	To determine if early vs. delayed ROM after RTSA affected postoperative ROM, PROM and the dislocation rate. Data was collected on ROM, ASES scores, and complications over 1-y follow-up.	Both early and delayed ROM after RTSA showed similar outcome improvements; early rehab may help elderly avoid issues from prolonged immobilization.	USA	RCT	Total: 86 Delayed: 44 Immediate: 42	Delayed: 50.0 Immediate: 69.0	Delayed: 69.4 ± 7.5 Immediate: 68.3 ± 10.5	Delayed: 28.9 ± 6.3 Immediate: 30.8 ± 7.3
Leas et al^34^ (2019)	Opioid-free shoulder arthroplasty: a prospective study of a novel clinical care pathway	Journal of Shoulder and Elbow Surgery	Compared opioid-sparing against opioids for perioperative pathway in patients undergoing elective shoulder arthroplasty. Data pain, opioid use, delirium, nausea, constipation, and falls were collected.	An opioid-sparing protocol is safe, effective, and rarely needs rescue opioids in well-selected shoulder arthroplasty patients.	USA	Prospective observational study	35	48.6	70.8 ± 8.9	29.3 ± 6.1
Denard and Lädermann^12^ (2016)	Immediate versus delayed passive range of motion following total shoulder arthroplasty	Journal of Shoulder and Elbow Surgery	Comparing immediate against delayed ROM following TSA. Data on ROM and functional outcomes was collected over 1-y follow-up.	Immediate ROM leads to quicker functional recovery, but there are no long-term differences in ROM or functional outcomes. Immediate ROM may reduce the healing rate of a lesser tuberosity osteotomy.	USA	RCT	Total: 55 Immediate: 27 Delayed: 28	Immediate: 44 Delayed: 61	Immediate: 69.1 Delayed: 66.9	
Garcia et al^18^ (2022)	Tranexamic acid in total shoulder arthroplasty under regional anesthesia: a randomized, single blinded, controlled trial	Braz J Anesthesiol.	Evaluating if TXA reduced perioperative blood loss in TSA. Data on blood loss, hemoglobin variation, recovery quality and coagulation was collected.	TXA reduced blood loss and hemoglobin drop in TSA.	Portugal	RCT	Total: 45 TXA: 23 NTXA: 22	15.6	76 ± 6.4	28.1 ± 4.0
Cheah et al^8^ (2022)	Orthopedic sleep and novel analgesia pathway: a prospective randomized controlled trial to advance recovery after shoulder arthroplasty	Journal of Shoulder and Elbow Surgery	Evaluating if a multimodal sleep pathway could improve patient analgesia after TSA. Multimodal sleep pathway included nonpharmacologic sleep measures, zolpidem, and melatonin.	The interventional sleep pathway improves analgesia, reduces opioid use, increases sleep duration, and enhances sleep quality during inpatient observation.	USA	RCT	Total: 125 Control: 64 Intervention: 61	Control: 54.7 Intervention: 47.6	Control: 66.3 ± 11.1 Intervention: 66.0 ± 9.07	Control: 28.7 ± 6.3 Intervention: 28.8 ± 5.1
Patel et al^47^ (2020)	Brachial Plexus Block with Liposomal Bupivacaine for Shoulder Surgery Improves Analgesia and Reduces Opioid Consumption: Results from a Multicenter, Randomized, Double-Blind, Controlled Trial	Pain Medicine	Comparing single-injection BPB with LB added to a placebo group. Primary outcome was pain scores over 48 h. Secondary outcomes were opioid consumption and adverse events.	BPB with LB provided analgesia for 48 h postsurgery, reducing opioid use compared to placebo after shoulder surgery.	USA	RCT	Total: 155 LB: 84 Placebo: 71	LB: 63.8 Placebo: 67.6	LB: 60.6 ± 9.9 Placebo: 58.5 ± 9.5	LB: 30.7 ± 4.6 Placebo: 30.2 ± 5.5
Jolissaint et al^28^ (2022)	Opioid-free shoulder arthroplasty is safe, effective, and predictable compared with a traditional perioperative opiate regimen: a randomized controlled trial of a new clinical care pathway	Journal of Shoulder and Elbow Surgery	Evaluating if an new opioid-sparing pain management pathway could improve outcomes for patients. Data was collected on pain scores, readmissions and opioid side effects.	An opioid-sparing multimodal pain management pathway is safe, effective, and provides superior pain relief compared to the traditional opioid-based approach at 12 h, 24 h, and 2 weeks postoperative.	USA	RCT	67	0.4	74.1 ± 6.7	28 ± 4.1
Panchamia et al^45^ (2019)	A 3-arm randomized clinical trial comparing interscalene blockade techniques with local infiltration analgesia for total shoulder arthroplasty	Journal of Shoulder and Elbow Surgery	Comparing CIB against SSIB and LIA for analgesic efficacy. Primary outcome was OBAS after 24 h. Secondary outcomes included pain scores, opioid use and complications.	CIB offers better analgesia than SSIB and local infiltration in the first 24 h after TSA.	USA	RCT	Total: 125 Single: 42 Continuous: 41 Local: 42	Single: 48 Continuous: 46 Local: 60	Single: 67.8 ± 13.1 Continuous: 68.1 ± 10.1 Local: 69.5 ± 8.9	Single: 30.3 ± 5.2 Continuous: 30.2 ± 5.7 Local: 30.9 ± 5.0
Rhyner et al^52^ (2024)	Single-bolus injection of local anesthetic, with or without continuous infusion, for interscalene brachial plexus block in the setting of multimodal analgesia: a randomized controlled unblinded trial	Reg Anesth Pain Med	Comparing efficacy of CIB against SSIB for BPB. Primary outcomes were cumulative morphine use at 24 h. Secondary outcomes were pain scores and functional outcomes over 48 h.	CIB did not provide better analgesia than a single injection in multimodal analgesia after shoulder surgery. Study limitations include performance and detection biases.	Switzerland	RCT	Total: 54 Continuous: 27 Single: 27	Continuous: 40.7 Single: 51.9	Continuous: 65.0 ± 10.0 Single: 64.0 ± 13.0	Continuous: 25.5 ± 4.6 Single: 28.7 ± 5.8
Pai et al^44^ (2025)	Comparison of analgesic efficacy of continuous perineural catheter, liposomal bupivacaine, and dexamethasone as an adjuvant for interscalene block in total shoulder arthroplasty: a triple-blinded randomized controlled trial	Journal of Shoulder and Elbow Surgery	Comparing LP to SSIB with added dexamethasone as analgesia. Group A had dexamethasone with bupivacaine, group B had LB and group C had ISB. Primary outcomes were pain scores, secondary outcomes were opioid use, hospital stay.	No significant difference in analgesia was found between any of the groups. Providers should choose the best option based on patient needs, as all 3 provide similar efficacy.	USA	RCT	Total: 72 Group A: 24 Group B: 24 Group C: 24	Group A: 41.7 Group B: 29.2 Group C: 45.8	Group A: 69.1 ± 6.2 Group B: 71.8 ± 6.6 Group C: 68.0 ± 8.5	
Sabesan et al^56^ (2017)	A prospective randomized controlled trial to identify the optimal postoperative pain management in shoulder arthroplasty: liposomal bupivacaine versus continuous interscalene catheter	Journal of Shoulder and Elbow Surgery	Comparing SSIB against CIB in regard to postoperative pain control. Data was collected on patient-reported outcomes and narcotic use.	LB gave strong pain relief with fewer complications and lower cost, supporting its use in multimodal pain management for shoulder arthroplasty.	USA	RCT	Total: 70 CISB: 36 LB: 34	CISB: 52.8 LB: 73.5	CISB: 65.0 LB: 63.0	
Mizels et al^39^ (2025)	Hydrogen Peroxide May Reduce the Risk for Revision Surgery and Infection in Primary Shoulder Arthroplasty: Two-yr Follow-up From a Prospective, Blinded, Controlled Trial	J Am Acad Orthop Surg	To determine if the addition of hydrogen peroxide to the preoperative skin preparation for TSA affected patient-reported outcomes. Data was collected on outcomes (ASES, SST, VAS), infection, and revision rates assessed at 2 y.	Hydrogen peroxide added to skin prep is safe; may reduce C. acnes infection and revision risk.	USA	Prospective, Blinded, Controlled Trial	Total: 61 Control: 31 Peroxide: 30	48% in both groups	Control: 67.6 ± 7.5 y, Peroxide: 69.2 ± 8.6 y	Control: 32.5 ± 7.3, Peroxide: 31.3 ± 9.4
Flurin et al^15^ (2023)	Outpatient vs. inpatient total shoulder arthroplasty: complication rates, clinical outcomes, and eligibility parameters	Journal of Shoulder and Elbow Surgery	To determine the postoperative outcomes of the TSA outpatient population. Data was collected on complications, readmissions, revisions, functional scores, and pain up to 12 mo.	Outpatient TSA was safe and independently linked to better early function.	France	Prospective Cohort Study	Total: 165 Outpaitent: 106 Inpatient: 59	Outpatient: 43 Inpatient: 29	Outpatient: 72.8 ± 6.6 Inpatient: 76.1 ± 6.3	
Castle et al^6^ (2024)	Implementation of a nonopioid multimodal analgesia protocol significantly reduces opioids prescribed after total shoulder arthroplasty: a retrospective study	Seminars in Arthroplasty Journal of Shoulder and Elbow Surgery	Comparing opioid-sparing approach against standard opioid regimen for analgesia effects. Data was collected on pain scores, PROMIS, and opioid use up to 3 mo.	Nonopioid regimen after TSA reduced opioid use with similar pain scores, outcomes, and no rise in complications	USA	Retrospective Case-Control Study	Total: 344 Opioid: 232 Multimodal: 112	Opioid: 50.4 Multimodal: 48.2	Opioid: 69.9 ± 8.5 Multimodal: 70.6 ± 8.1	Opioid: 29.6 ± 6.1 Multimodal: 31.0 ± 5.9
Jones et al^29^ (2022)	Opioid-Sparing Pain Management Protocol After Shoulder Arthroplasty Results in Less Opioid Consumption and Higher Satisfaction: a Prospective, Randomized Controlled Trial	Journal of shoulder and elbow surgery	Comparing opioid-sparing approach against standard opioid regimen for analgesia effects. Data was collected on pain scores, opioid use, ROM, ASES, and SANE scores up to 12 weeks.	Opioid-sparing protocol cut opioid use nearly 4-fold, led to earlier cessation, and improved satisfaction without affecting outcomes.	USA	RCT	Total: 78 OB: 42 OS: 36	OB: 64 OS: 56	OB: 72.4 ± 5.4 OS: 72.1 ± 6.6	OB: 30.2 ± 6.3 OS: 30.5 ± 4.1
Panchamia et al^46^(2025)	Randomized Clinical Trial Comparing Mixed Liposomal Bupivacaine versus Continuous Catheter for Interscalene Block during Shoulder Arthroplasty: a Comparison of Analgesia, Patient Experience, and Cost	Journal of shoulder and elbow surgery	Comparing CIB against SSIB in regards in regard to analgesic efficacy. Primary outcome was pain scores on POD 1. Secondary outcomes include opioid use, complications, and recovery on POD 3.	Single-injection ISB with LB gave similar analgesia to CIB through POD 3, with better overall value.	USA	RCT	Continuous: 83 Single-Injection: 39	55.40%	Continuous: 72.0 ± 7.3 Single-Injection: 70.9 ± 7.3	Continuous: 29.4 ± 6.1 Single-Injection: 28.0 ± 5.6
Schumaier et al^57^(2023)	Interscalene block vs. periarticular liposomal bupivacaine for pain control following reverse shoulder arthroplasty: a randomized trial	Journal of Shoulder and Elbow Surgery	Comparing the effects of preoperative interscalene nerve block against intra-op periarticular injection. Data was collected on narcotic consumption, pain scores, and satisfaction. Secondary outcomes included psychosocial factors, LOS, and readmissions.	The Block group had lower narcotic use and higher satisfaction, with no significant differences in pain scores, stay length, or readmissions. Psychosocial factors and patient preferences did not affect satisfaction.	USA	RCT	Total: 76 Block: 38 Local: 38	55.3	67.5	32.55
Sun et al^63^ (2022)	Continuous interscalene versus phrenic nerve-sparing high-thoracic erector spinae plane block for total shoulder arthroplasty: a randomized controlled trial	Canadian Journal of Anesthesia/Journal canadien d'anesthésie	Comparing HT-ESPB against an interscalene block for TSA. Primary outcomes included incidence of hemidiaphramatic paralysis. Secondary outcomes included adverse events, pain scores, and opioid consumption.	Interscalene provided better opioid-sparing efficacy in the immediate postoperative period. Further studies with larger sample sizes are needed to assess analgesic outcomes and recovery.	USA	RCT	Total: 26 Interscalene: 14 HT-ESPB: 12	Interscalene: 71 HT-ESPB: 58	Interscalene: 70.4 ± 12.4 HT-ESPB: 72.3 ± 6.5	Interscalene: 26.3 ± 2.3 HT-ESPB: 27.8 ± 3.9

*BMI*, body mass index; *SD*, standard deviation.

### Study characteristics

The mean age of patients across the included studies ranged from approximately 58 to 76 years. Gender representation varied considerably, with the proportion of male participants ranging from 0.4% to 73.5%. Mean body mass index, where reported, ranged from 25.5 to 32.5 kg/m^2^. Sample sizes differed substantially, ranging from 26 to 344 patients across studies (Table I).

With respect to procedure type, anatomic TSA was the most frequently studied procedure (n = 7), while only 2 studies focused exclusively on RTSA. The remaining studies included mixed SA populations (TSA and RTSA) without stratification by implant type (n = 9), limiting procedure-specific conclusions (Table I).

Geographically, the majority of studies were conducted in the United States (n = 15), with additional contributions from France (n = 1), Portugal (n = 1), and Switzerland (n = 1). Most studies were RCTs, with fewer prospective cohorts and retrospective observational and case–control designs represented (Table I).

## Analgesic and rehabilitation protocols

Table II summarizes the analgesic and rehabilitation strategies used across the 18 included studies. The protocols varied considerably, reflecting differences in anesthetic technique, multimodal pain management, OS strategies, and postoperative rehabilitation plans following SA. Most studies incorporated regional anesthesia (eg, interscalene blocks, continuous or single shot), multimodal analgesia with non-steroidal anti-inflammatory drugs (NSAIDs), acetaminophen, and adjuncts such as gabapentin or dexamethasone, and clearly defined postoperative rehabilitation pathways. OS approaches were explicitly employed in over half of the studies (n = 10), highlighting a contemporary shift toward minimizing opioid exposure while maintaining effective analgesia.

**Table II:** Summary of included studies reporting analgesic and rehabilitation protocols following shoulder arthroplasty.

Authors (yr)	Type of anesthesia used	Preoperative pain management	Intra-operative pain management	PACU (postanesthesia care unit)	Postoperative medications	Discharge medications	Use of regional anesthesia/Nerve blocks (%)	Multimodal analgesia protocols use	Opioid-sparing strategies (yes/no)	Postoperative rehabilitation plan (standardized vs. Nonstandardized)	Pain management strategy used (opioid-based, NSAIDs, etc.)
Hagen et al^24^ (2020)										Immediate ROM and Delayed ROM (by 6 weeks)	
Leas et al^34^ (2019)	GA and ISB	Gabapentin, Celecoxib, ISB	Acetaminophen LB, nonopioid anesthetics (fluranes, propofol)	Ketorolac, and Cryotherapy	Maintenance of cryotherapy, Ketorolac, Celecoxib, Gabapentin, Acetaminophen	Gabapentin, Celecoxib, Acetaminophen	Yes	Yes	Yes		NSAIDs, gabapentin, and cryotherapy
Denard and Lädermann^12^ (2016)										Immediate ROM and Delayed ROM	
Garcia et al^18^ (2022)	ISB		Ropivacaine, Lidocaine, and Dexamethasone, and Propofol sedation		Paracetamol, Tramadol, Ketorolac, Dipyrone, Morphine	Enoxaparin, compression stockings	Yes	Yes	Yes		Opioid and NSAIDs
Cheah et al^8^ (2022)	GA and ISB	Block group: ISB using ropivacaine. Local group: No preoperative pain management mentioned	Block group: ISB with ropivacaine. Local group: IB		Oxycodone, Acetaminophen, Ibuprofen, Gabapentin		Yes	Yes	Yes	Standardized	Opioids, NSAIDs
Patel et al^47^ (2020)	GA and SSIB	Celecoxib, Gabapentin, Acetaminophen	ISB with Ropivacaine		Naproxen, Gabapentin, Acetaminophen, Oxycodone, Hydromorphone		Yes	Yes	Yes		Opioids, NSAIDs, and pharmacologic sleep interventions
Jolissaint et al^28^ (2022)	ISB	Peripheral nerve catheter with IV sedation (fentanyl and midazolam). Ropivacaine administered after catheter placement.	Ropivacaine, opioids (fentanyl or hydromorphone)	ISC with Ropivacaine	Acetaminophen, Gabapentin, Oxycodone, Hydromorphone	Continued multimodal analgesia with discharge	Yes	Yes	Yes	Standardized	Opioids, NSAIDs, and ISB
Panchamia et al^45^ (2019)	BPB with LB	Acetaminophen	Fentanyl, Sufentanil, Remifentanil, Ketorolac, or NSAIDs		Acetaminophen, Oxycodone, Morphine, Hydromorphone	Acetaminophen and Oxycodone	Yes	Yes	Yes	Standardized	Opioids, NSAIDs, and ISB
Rhyner et al^52^ (2024)	ISB and nonopioid GA	ISB, Meloxicam, Gabapentin, Acetaminophen, Scopolamine patch	Propofol, Sevoflurane LB, Dexamethasone, Morphine, Hydromorphone, Fentanyl	Cryotherapy, Ketorolac, Hydrocodone-acetaminophen	Cryotherapy. Gabapentin, Ketorolac, Hydrocodone-acetaminophen	Cryotherapy, Meloxicam, Gabapentin, Oxycodone-Acetaminophen, Aspirin	Yes	Yes	Yes	Standardized	Opioids, NSAIDs, and cryotherapy
Pai et al^44^ (2025)	GA	Acetaminophen, Celecoxib, Oxycodone	Dexamethasone	Fentanyl or Hydromorphone, Acetaminophen, Ketamine, Oxycodone	Acetaminophen, Oxycodone, Hydromorphone, Fentanyl	Oxycodone and Acetaminophen	Yes	Yes	Yes	Nonstandardized	Opioids, NSAIDs
Sabesan et al^56^ (2017)	BPB	ISB and Midazolam	Dexamethasone, Ketorolac, Acetaminophen	57	Acetaminophen, Ibuprofen	No	Yes	Yes	Yes	Standardized	Opioids and ISB
Mizels et al^39^ (2025)	GA and ISB		Midazolam, Fentanyl and ISB.		Oxycodone, Hydromorphone, Acetaminophen		Yes	Yes	Yes		Opioids, NSAIDs and ISB
Flurin et al^15^ (2023)	GA	Acetaminophen, Celecoxib	Fentanyl and LB		Celecoxib, Oxycodone/Acetaminophen, Morphine		Yes	Yes	Yes		Opioids, NSAIDs and ISB
Castle et al^6^ (2024)											
Jones et al^29^ (2022)	GA and ISB	ISB	SSIB and TXA			Multimodal analgesia protocol continued Opioid-sparing strategy used	Yes	Yes	Yes	Standardized	NSAIDs and opioid-sparing analgesia
Panchamia et al^46^ (2025)	GA	Celecoxib, Pregabalin, Tramadol	Ropivacaine, Epinephrine, Ketorolac, Dexamethasone, Acetaminophen		Dexamethasone, Pregabalin, Tizanidine, Ibuprofen, Oxycodone/Acetaminophen	Dexamethasone, Pregabalin, Tizanidine, Magnesium, Ibuprofen, Oxycodone, Acetaminophen	No	Yes	Yes	Standardized	Opioids and NSAIDs
Schumaier et al^57^ (2023)		Acetaminophen, Gabapentin, Celecoxib. Opioid-based group received an additional preoperative dose of oxycodone	LB and Ketorolac		Acetaminophen, Gabapentin, Ketorolac, Oxycodone, Celecoxib, Hydromorphone	Oxycodone Acetaminophen, Gabapentin, Celecoxib, Cryotherapy		Yes	Yes	Standardized	Opioids, NSAIDs, and cryotherapy
Sun et al^63^ (2022)	GA	Acetaminophen, Celecoxib, Scopolamine, Caffeine	Dexamethasone, Opioids (Fentanyl, Hydromorphone, Ketorolac, Ketamine)	Fentanyl and Hydromorphone, Acetaminophen, Meloxicam	Acetaminophen, Meloxicam, Fentanyl, Hydromorphone, Oxycodone	Acetaminophen, Meloxicam, Fentanyl, Hydromorphone, Oxycodone	Yes	Yes	Yes		Opioids and NSAIDs

*NSAIDs*, non-steroidal anti-inflammatory drugs.

### Risk of bias

The risk of bias was assessed using the ROBINS-I tool for nonrandomized studies (n = 4) and the RoB 2 tool for RCTs (n = 14; Figure 2, Figure 3).

Among the nonrandomized studies (non-RCTs), 75% were judged to have a moderate overall risk of bias, while 25% had a serious risk. In terms of specific domains, bias due to confounding was rated as low in 25% of studies and moderate in 75%. Bias due to deviations from intended interventions and bias in outcome measurement were both evenly split, with 50% of studies rated as low risk and 50% as moderate risk. Bias in the classification of interventions and bias due to missing data each demonstrated a low risk in 75% of studies and a moderate risk in 25%. Notably, all studies (100%) were rated as low risk for bias in the selection of participants. Conversely, all studies exhibited a moderate risk of bias in the selection of the reported results.

Among the RCTs, 14.3% were assessed as having a low overall risk of bias, 64.3% as having some concerns, and 21.4% as having a high risk. Most domains demonstrated a predominance of low or moderate risk. Bias due to missing outcome data was the most robust, with all studies rated as low risk. Bias arising from the randomization process and bias in the selection of the reported results were also generally low, with 78.6% and 85.7% of studies respectively receiving low-risk ratings. In contrast, bias due to deviations from intended interventions showed more concern, with only 21.4% rated as low risk and the remaining 78.6% flagged for some concerns. Similarly, bias in the measurement of the outcome was rated low in 21.4%, with 71.4% having moderate concerns and 7.1% considered as high risk of bias.

### Clinical outcomes

Table III summarized key clinical outcomes reported in the included studies. These outcomes included functional PROMs, ROM and patient satisfaction. The data presented provided a comprehensive overview of the clinical efficacy of different protocols, highlighting notable trends, differences, and outcomes in the postoperative management of SA patients.

**Table III:** PROMs and patient satisfaction.

Authors (yr)	American Shoulder and Elbow Surgeons (ASES) score	Range of motion (°)	Simple Shoulder Test (SST) score	Single Assessment Numeric Evaluation (SANE) score	Quality of Recovery-15 (QoR-15) scores	Other PROMS data	Satisfaction scores (Likert scale, % satisfied)
Hagen et al^24^ (2020)	Preoperative -Delayed: 38.5 ± 18.9 Immediate: 44.2 ± 17.4 6 mo - Delayed: 40.2 ± 20.1 Immediate: 30.0 ± 18.8						
Leas et al^34^ (2019)	Preoperative: 52.5 2 weeks: 54.2 2 mo: 71.6		Preoperative: 5.0 (IQR: 3.0 - 6.0) 2 mo: 6.0 (IQR: 4.0 - 7.0)			VR-12: Preop: 59.4 (IQR: 51.7 - 62.7), 2-mo: 60.8 (IQR: 49.4 - 64.0)	2 weeks: 97.1 2-mo: 93.5
Denard and Lädermann^12^ (2016)	Preoperative - Immediate: 34.0 ± 11.3, Delayed: 39.4 ± 18.2 1 yr - Immediate: 89.0 ± 10.9, Delayed: 88.9 ± 13.1	Forward flexion - Preoperative: Immediate - 106 ± 34, Delayed - 104 ± 28 1 y: Immediate - 142 ± 20, Delayed - 146 ± 20 External rotation -Preoperative: Immediate - 21 ± 16, Delayed - 20 ± 16 1 y: Immediate - 62 ± 16, Delayed - 57 ± 12	Preoperative: Immediate: 3.1 ± 2.2, Delayed: 3.7 ± 2.6 1-y: Immediate: 9.9 ± 2.5, Delayed: 9.8 ± 2.4	Preoperative: Immediate: 32.7 ± 23.5, Delayed: 38.2 ± 24.9 1-y: Immediate: 85.8 ± 20.6, Delayed: 88.2 ± 12.4			
Garcia et al^18^ (2022)					TXA: 128.2 ± 22.3 NTXA: 121.4 ± 23.1		
Cheah et al^8^ (2022)						LSEQ Ease of Going to Sleep (mean): Control: 39.9 ± 15.8, Interventional: 45.1 ± 11.3 Quality of Sleep (mean): Control: 34.4 ± 18.4, Interventional: 36.3 ± 17.7 Ease of Awakening (mean): Control: 50.0 ± 16.8, Interventional: 47.1 ± 17.3	Satisfaction scores with pain: Intervention: 89.2, Control: 79.6 Satisfaction scores with sleep: Intervention: 82.1, Control: 76.8
Patel et al^47^ (2020)							Satisfaction with overall analgesia: 1 d: LB: 4.4 ± 0.81, Placebo: 3.7 1.32 10 d: 4.5 ± 0.95, Placebo: 4.0 ± 1.15
Jolissaint et al^28^ (2022)						PACSYM Preoperative: OB: 2 (IQR: 0.0 - 6.5), OS: 1 (IQR: 0.0 - 7.0) 6 weeks: OB: 0.0 (IQR: 0.0 - 5.0), OS: 1.5 (IQR: 0.0 - 6.0)	2 weeks: OB: 90.6, OS: 94.3 6 weeks: OB: 86.7, OS: 97.1
Panchamia et al^45^ (2019)	Preoperative - Local: 44.0 ± 19.7, SSIB: 52.8 ± 17.7, CIB: 42.0 ± 15.4 Follow-up - Local: 89.7 ± 14.3, SSIB: 90.3 ± 10.6, CIB: 89.9 ± 13.4					SF-12: Physical Composite Scale Score - Preoperative: LIA: 35.0 ± 9.6, SSIB: 36.4 ± 8.0, CIB: 35.9 ± 8.7 3 mo: LIA: 42.0 ± 9.6, SSIB: 43.7 ± 8.6, CIB: 42.1 ± 9.3 Mental Composite Scale Score - Preoperative: LIA: 54.0 ± 9.6, SSIB: 55.3 ± 9.6, CIB: 55.4 ± 8.6 3 mo: LIA: 56.3 ± 7.6, SSIB: 55.5 ± 8.1, CIB: 59.1 ± 5.1	
Rhyner et al^52^ (2024)		Forward Flexion (1-d postoperative)- CIB: 40 (IQR: 30 - 68), SSIB: 45 (IQR: 35 - 80) External rotation (1-d postoperative) 1 d - CIB: 0 (IQR: -25 - 0), SSIB: 0 (IQR: -10 - 0)					CIB: 100, SSIB: 100
Pai et al^44^ (2025)						PROMIS Assessment Physical: Preoperative: Group A: 41.98 ± 9.88, Group B: 42.42 ± 8.83, Group C: 41.19 ± 9.26 1 week: Group A: 44.44 ± 9.57, Group B: 43.55 ± 9.52, Group C: 45.15 ± 8.89 Mental: Preoperative: Group A: 50.00 ± 12.18, Group B: 51.64 ± 11.01, Group C: 51.44 ± 8.35 1 week: Group A: 49.75 ± 11.51, Group B: 52.14 ± 10.41, Group C: 52.99 ± 6.93	Group A: 66.7 Group B: 66.7 Group C: 66.7
Sabesan et al^56^ (2017)	Preoperative - CIB: 27.7 ± 15.7, LB: 36.1 ± 20.4 6 weeks: CIB: 57.3 ± 16.2, LB: 59.5 ± 18.1	Forward Flexion: 6 weeks: CIB: 103, LB: 92 12 weeks: CIB: 96, LB: 130 External Rotation: 6 weeks: CIB: 30, LB: 22 12 weeks: CIB: 29, LB: 32				Penn Shoulder Score: Preoperative: CIB: 19.4 ± 11.7, LB: 25.8 ± 15.7 6 weeks: CIB: 50.7 ± 22.5, LB: 52.7 ± 22.6 Subjective Shoulder Value: Preoperative: CIB: 24.3 ± 23.4, LB: 30.7 ± 22.7 6 weeks: CIB: 63.1 ± 19.1, LB: 55.7 ± 24.6	Satisfaction score out of 5: Day 2: CIB: 4.3 ± 0.9, LB: 4.3 ± 1.1 Day 30: CIB: 4.5 ± 0.8, LB: 4.6 ± 0.7
Mizels et al^39^ (2025)	Control Group: 84 ± 17 Peroxide Group: 85 ± 19		Control Group: 10.1 ± 2.0 Peroxide Group: 10.0 ± 2.3				
Flurin et al^15^ (2023)	Preoperative - Inpatient: 32.5 ± 16.6, Outpatient: 37.5 ± 15.9 6 mo - Inpatient: 78.6 ± 18.9, Outpatient: 74.6 ± 21.2 12 mo - Inpatient: 83.2 ± 17.0, Outpatient: 85.2 ± 14.7					Functional Outcome Measure for Shoulder: Preoperative: Inpatient: 36.6 ± 14.2, Outpatient: 39.7 ± 13.2 12 mo: Inpatient: 73.3 ± 8.9, Outpatient: 77.5 ± 9.2 Shoulder Pain and Disability Index: Preoperative: Inpatient: 83.2 ± 31.6, Outpatient: 79.4 ± 22.3 12 mo: Inpatient: 25.8 ± 27, Outpatient: 18.9 ± 20.7	1.5 mo: Inpatient: 86.5, Outpatient: 91.3
Castle et al^6^ (2024)						PROMIS-UE (Upper Extremity): Preoperative: OB: 28.7 ± 6.1, OS: 29.0 ± 7.0 3 mo: OB: 37.1 ± 6.7, OS: 36.3 ± 6.4 PROMIS-PI (Pain Interference): Preoperative: OB: 63.0 ± 7.4, OS: 63.0 ± 7.0 3 mo: OB: 53.4 ± 8.6, OS: 55.5 ± 6.8 PROMIS-D (Depression): Preoperative: OB: 49.9 ± 13.1, OS: 48.0 ± 11.0 3 mo: OB: 45.5 ± 22.4, OS: 46.0 ± 22.0	
Jones et al^29^ (2022)	Preoperative - OB group: 40 ± 20, OS group: 38 ± 19 12 weeks - OB group: 69 ± 24, OS group: 71 ± 25	Forward Flexion: Preoperative: OB - 84 ± 39, OS - 91 ± 48 12 weeks: OB - 126 ± 26, OS - 123 ± 28 External Rotation: Preoperative: OB - 29 ± 20, OS - 30 ± 21 12 weeks: OB - 39 ± 12, OS - 41 ± 214		Preoperative: OB: 42 ± 25, OS: 35 ± 23			1 Week: OB: 88 ± 21, OS: 95 ± 13 12 Weeks: OB: 93 ± 14, OS: 94 ± 15
Panchamia et al^46^ (2025)					POD 1: CIB: 124.0 (IQR: 111.0 - 132.5), LB: 126.0 (IQR: 113.0 - 133.0		
Schumaier et al^57^ (2023)	Preoperative - Local: 44.0 ± 23.7, SSIB: 52.8 ± 17.7, CIB: 42.0 ± 15.4 Follow-up - Local: 89.7 ± 14.3, SSIB: 90.3 ± 10.6, CIB: 89.9 ± 13.4					Pain Catastrophizing (mean): Block: 11.9 ± 9.3, Local: 9.1 ± 9.3 Resilience (mean): Block: 3.9 ± 0.7, Local: 3.8 ± 0.6	Block: 83 ± 23 Local: 68 ± 31
Sun et al^63^ (2022)						Hemi diaphragmatic Paralysis (%): ISB: None: 0%, Partial: 36, Full: 64, HT-ESPB: None: 100%, Partial: 0, Full: 0 Percent Decrease in ISV from Baseline (%): ISB: PACU: 43.7 ± 17.1, POD 1: 37.9 ± 17.1, HT-ESPB: PACU: 9.0 ± 8.1, POD 1: 6.6 ± 7.6	ISB: 100 HT-ESPB: 100

*ROM*, range of motion; *RTSA*, reverse total shoulder arthropathy; *PROM*, patient-reported outcomes measures; *ASES*, American Shoulder and Elbow Surgeons; *RCT*, randomized controlled trial; *TSA*, total shoulder arthropathy; *TXA*, tranexamic acid; *BPB*, brachial plexus block; *LB*, liposomal bupivacaine; *CISB*, continuous interscalene block; *SSIB*, single-shot interscalene blockage; *LIA*, local infiltration analgesia; *ISB*, interscalene blockage; *SST*, simple shoulder test; *VAS*, visual analog scale; *PROMIS*, Patient-Reported Outcome Measurement Information System; *SANE*, Single Assessment Numeric Evaluation; *POD*, postoperative day; *LOS*, length of stay; *HT-ESPB*, high-thoracic erector spine plane block; *OB*, opioid based; *OS*, opioid sparing; *GA*, general anesthesia; *VR-12 MC*, Veterans RAND 12-item Mental Component; *LSEQ*, Leeds Sleep Evaluation Questionnaire; *PACSYM*, Patient Assessment of Constipation Symptoms; *SF-12*, Medical Outcomes Study 12-item Short Form; *IQR*, interquartile range.

## Functional patient-reported outcome measures (PROMs)

### ASES score

Eight studies reported ASES scores as a primary indicator of postoperative recovery and functional improvement following SA.^11^^,^^14^^,^^23^^,^^28^^,^^34^^,^^39^^,^^46^^,^^56^ Hagen *et al*^23^ compared early versus delayed rehabilitation following RTSA and demonstrated that delayed mobilization led to significantly greater improvements in ASES pain scores at six months (26.3 ± 16.3 vs. 16.7 ± 11.6; *P* = .01), along with higher overall ASES scores (40.2 ± 20.1 vs. 30.0 ± 18.8; *P* = .04). In contrast, Denard and Lädermann^11^ investigated immediate versus delayed passive ROM protocols and found no statistically significant difference in 1-year ASES scores (immediate: 89.0 ± 10.9 vs. delayed: 88.9 ± 13.1; *P* = .4), suggesting that long-term functional outcomes were similar between these approaches.

Leas *et al*^34^ evaluated the effect of an opioid-free perioperative protocol on ASES subscales and reported a gradual improvement in both activities of daily living (ADL) and function components over two months. While ASES ADL scores initially dropped from 13.0 (interquartile range 9.0-16.0) to 5.0 (2.0-9.0) at two weeks, they rebounded to 14.0 (11.0-19.0) by two months, indicating a recovery trend; similarly, ASES Function scores improved from 52.5 (43.3-65.0) to 71.6 (63.8-78.3), although no statistical tests were reported.^34^ Panchamia *et al*^46^ compared 3 analgesic techniques local infiltration analgesia (LIA), single-injection interscalene block, and continuous interscalene block (CISB) and found that all groups experienced significant postoperative improvements in ASES scores, with the CISB group demonstrating the most consistent gains. Notably, the change in ASES scores from baseline to follow-up was statistically significant across all groups (*P* = .02).^46^

Sabesan *et al*^56^ compared LB with CISB and found that final ASES scores were significantly higher in the LB group (74.5 ± 20.5 vs. 59.7 ± 22.7; *P* = .02), although no significant differences were observed at the 6-week timepoint. Mizels *et al*^39^ investigated whether adding hydrogen peroxide to preoperative skin preparation affected functional outcomes yet found no significant differences in total ASES scores between groups at two years (control: 84 ± 17 vs. Peroxide: 85 ± 19; *P* = .7), nor in pain or ADL subdomains. Similarly, Flurin *et al*^14^ reported no significant baseline ASES score differences between inpatient and outpatient groups; while a significant improvement at 1.5 months was observed in the outpatient cohort (70.4 ± 18.6 vs. 60.4 ± 20.3; *P* = .01), these gains were not sustained at 6 or 12 months. Lastly, Jones *et al*^28^ compared opioid-based (OB) and OS analgesia and noted no significant differences in ASES scores, either preoperatively (OB: 40 ± 20 vs. OS: 38 ± 19) or at 12 weeks postoperatively (OB: 29 ± 24 vs. OS: 33 ± 25).

### ROM

Four studies evaluated postoperative ROM as a key functional outcome following SA.^11^^,^^28^^,^^52^^,^^56^ Denard and Lädermann^11^ compared immediate versus delayed rehabilitation and reported no significant differences at 1-year follow-up in forward flexion (142° ± 20 vs. 146° ± 20; *P* = .9), external rotation (62° vs. 57°; *P* = .2), or internal rotation (L3 vs. L1 spinal level; *P* = .7), indicating both protocols offered comparable motion recovery. Similarly, Rhyner *et al*^52^ compared single-shot with CISBs and found no meaningful differences in active-assisted forward flexion, abduction, or external rotation at any postoperative time point.

Sabesan *et al*^56^ noted temporal differences between the LB and CISB groups; for instance, at 12 weeks, forward flexion was greater in the LB group (130° vs. 96°; *P* = .07), whereas at 6 weeks, the CISB group showed better abduction (90° vs. 56°; *P* = .08), though these findings did not reach statistical significance. Moreover, Jones *et al*^28^ found no significant differences in ROM outcomes between OB and OS groups, with forward elevation at 12 weeks measuring 126° (standard deviation: 26) in the OB group versus 123° (standard deviation: 28) in the OS group (*P* = .8), and no meaningful variation in external or internal rotation from baseline to follow-up.

### SST score

Three studies evaluated the SST score in the context of ERAS-related interventions.^11^^,^^34^^,^^39^ Leas *et al*^34^ observed that patients undergoing an opioid-free protocol had a transient decline in SST scores at two weeks (median: 2.0, interquartile range 1.0-4.0), which subsequently improved to 6.0 (4.0-7.0) by two months, compared to a baseline of 5.0 (3.0-6.0); however, statistical significance was not reported. Denard and Lädermann found no significant differences between immediate and delayed rehabilitation in SST outcomes, with nearly identical 1-year scores (Immediate: 9.9 ± 2.5 vs. Delayed: 9.8 ± 2.4; *P* = .4), further supporting functional equivalence between these strategies.^11^ Similarly, Mizels *et al*^39^ found no differences in SST scores between the hydrogen peroxide and control groups, indicating that the skin preparation method did not influence this outcome metric.

### SANE score

Two studies assessed SANE scores following SA.^11^^,^^28^ Denard and Lädermann reported no significant difference between immediate and delayed ROM groups at 1-year follow-up (85.8 ± 20.6 vs. 88.2 ± 12.4; *P* = .9), suggesting that patient-reported global shoulder function was unaffected by timing of rehabilitation.^11^ Likewise, Jones *et al*^28^ compared OB and OS groups and found no significant difference in SANE score improvements at 12 weeks (change-from-baseline: 28 ± 28 vs. 24 ± 26), reinforcing the finding that analgesic strategy may not significantly alter perceived recovery.

### QoR-15

Two studies reported on QoR-15 as a composite measure of perioperative recovery quality^17^^,^^47^ Garcia *et al*^17^ compared TXA with non-TXA use and found no significant difference in QoR-15 scores (128.2 ± 22.3 vs. 121.4 ± 23.1; *P* = .4), indicating that TXA administration did not significantly affect early subjective recovery quality. Panchamia *et al*^47^ compared LB with CISBs and noted no significant differences on postoperative day 1 or 2, though by postoperative day 3, there was a trend toward improved QoR-15 scores in the LB group (132.0 vs. 124.5; *P* = .06), suggesting potential late-stage benefits in patient-perceived recovery.

### Patient satisfaction

Eleven studies assessed satisfaction following SA across different analgesic modalities.^7^^,^^14^^,^^27^^,^^28^^,^^34^^,^^45^^,^^48^^,^^52^^,^^56^^,^^57^^,^^63^

### Opioid-free vs. opioid-based pathways

Leas *et al*^34^ reported high levels of patient satisfaction using a fully opioid-free perioperative pathway, with 97.1% satisfied at 2 weeks and 93.5% at 2 months postoperatively. Jolissaint *et al*^27^ found no significant difference in 2-week satisfaction between opioid-free (94.3%) and opioid-containing (90.6%) groups (*P* = .5), but at 6 weeks, satisfaction was significantly higher in the opioid-free group (97.1% vs. 86.7%, *P* = .04). Jones *et al*^28^ compared OS and OB regimens, showing significantly greater satisfaction in the OS group at weeks 1 and 6 (*P* = .05 for both), but not at weeks 2 or 12.

### Liposomal bupivacaine (LB) vs. comparators

Mixed findings were reported for LB. Schumaier *et al*^57^ demonstrated significantly higher satisfaction scores with interscalene block versus LB (8.3 ± 2.3 vs. 6.8 ± 3.1, *P* = .02). Conversely, Patel *et al*^48^ found that LB significantly improved satisfaction compared to placebo at 24 hours (4.4 vs. 3.7, *P* = .002), 72 hours (4.5 vs. 4.1, *P* = .003), and 10 days (4.5 vs. 4.0, *P* < .001). Sabesan *et al*^56^ reported insignificant differences in satisfaction between CISB and LB at days 2, 7, or 30. Similarly, Pai *et al*^45^ found equivalent satisfaction across 3 analgesic approaches (LB, interscalene catheter, and dexamethasone-enhanced blocks), with 66.7% satisfaction in each group (*P* = 1).

### Other interventions

Rhyner *et al*^52^ observed no significant difference in satisfaction between continuous infusion and single-injection interscalene blocks (visual analog scale median: 10 vs. 10; *P* = .6). Cheah *et al*^7^ assessed a multimodal sleep pathway and reported no significant improvements in satisfaction with pain (89.2% vs. 79.6%, *P* = .2) or sleep management (82.1% vs. 76.8%, *P* = .5). Sun *et al*^63^ showed universal satisfaction (ie, 100%) with both continuous interscalene and high-thoracic erector spinae plane blocks (*P* = 1). Finally, Flurin *et al*^14^ found no significant difference in satisfaction between inpatient and outpatient arthroplasty at 1.5 months (91.3% vs. 86.5%), with similar outcomes at 6 and 12 months.

## Discussion

### Principal findings

This systematic review synthesized data from 18 studies involving 1,722 patients who underwent SA and received diverse analgesic and rehabilitation protocols. Across interventions, postoperative outcomes including ASES, SST, SANE, and QoR-15 scores generally improved, indicating that multiple strategies may achieve satisfactory functional and patient-reported outcomes. Notably, OS pathways and ERAS protocols were associated with high patient satisfaction and similar long-term functional recovery, with minimal differences in ROM.

### Contextualizing ERAS outcomes within the existing literature

#### Functional recovery and ASES trajectories

Large cohort studies show that recovery after SA follows a predictable trajectory, with most functional gains occurring within the first year and plateauing thereafter. Liu *et al*^35^ reported that 91.7% of patients achieved the minimal clinically important improvement and 70.4% achieved substantial clinical benefit by two years, with final ASES scores clustering in the high 80s to low 90s. Wong *et al*^71^ further demonstrated that poorer preoperative ASES scores (<25 pain; <12 function) were associated with larger postoperative gains, indicating that baseline status and biological healing exert greater influence on final outcomes than perioperative pathway variation. Together, these findings suggest that ERAS strategies are more likely to affect the rate and experience of recovery than ultimate functional endpoints.

#### Rehabilitation timing and range of motion

Evidence indicates that early rehabilitation may accelerate short-term motion without improving long-term ROM. Edwards *et al*^13^ found greater forward flexion at three months following early mobilization after RTSA (*P* = .02), but no difference at 12 months. Similarly, Lamb *et al*^33^ reported equivalent final ROM across protocols, alongside a higher incidence of acromial and scapular spine fractures in early mobilization cohorts. These data suggest that soft-tissue healing and implant biomechanics ultimately constrain final ROM, explaining why delayed or criteria-based rehabilitation does not compromise long-term motion.

#### Global functional PROMs (SST and SANE)

External validation studies consistently show that SST and SANE reflect overall functional recovery rather than protocol-specific effects. Godfrey *et al*^20^, ^54^ reported a standardized response mean of 0.81 for SST, with strong correlation to ASES (r = 0.80), while Roy *et al*^20^, ^54^ demonstrated an SRM exceeding 1.7, with clinically important change approximating 3 points. For SANE, correlations with ASES range from 0.77-0.82, with large effect sizes reported across shoulder populations.^2^^,^^69^ These characteristics likely explain why SST and SANE outcomes converge across differing ERAS strategies in the literature.

#### Quality of Recovery and early postoperative experience

QoR-15 captures early postoperative wellbeing and appears more sensitive to analgesic quality than rehabilitation structure. Clemens *et al*^12^ reported return to baseline QoR-15 within 48 hours following SA using multimodal analgesia. In contrast, Tan *et al*^64^ observed no significant QoR-15 improvement despite enhanced recovery pathways in hip arthroplasty, highlighting the multifactorial nature of early recovery. These findings suggest that QoR-15 differences are influenced by pain, sleep, nausea, and expectations rather than functional recovery alone.

#### Patient satisfaction and opioid stewardship

Across ERAS studies, patient satisfaction is more closely linked to opioid minimization than to functional superiority. Morgan *et al*^42^ demonstrated reduced length of stay (2.4 to 1.9 nights) and increased same-day discharge (40% to 49%) without adverse outcome effects. YaDeau *et al*^74^ reported higher satisfaction scores with ERAS (9.2 ± 1.7 vs. 8.2 ± 2.5; *P* < .001) alongside lower opioid consumption (23 ± 28 mg vs. 44 ± 35 mg; *P* < .01). These data suggest that reductions in opioid-related side effects, rather than differences in shoulder function, primarily drive patient satisfaction gains.

#### Clinical relevance

This review highlights key strategies to enhance recovery after SA, advocating a shift toward individualized, OS care. Based on current evidence, delayed mobilization following RTSA can be considered for patients at risk of soft-tissue compromise to optimize mid-term pain and function, whereas immediate rehabilitation may be safe for TSA patients with preserved bone quality, without compromising long-term ROM or functional recovery.^40^^,^^66^ Clinicians may therefore implement a protocolized approach stratified by procedure type and patient-specific risk factors, rather than a purely generic or ad hoc rehabilitation plan.

Equally, OS and opioid-free pathways produced comparable or superior functional outcomes and significantly higher patient satisfaction. This underscores the value of adopting multimodal analgesia as standard practice, positioning opioid minimization not as an adjunct but as a central pillar of recovery. Analgesic technique selection whether CISB or LB should be guided by patient comorbidities, institutional expertise, and resource availability, rather than pursuit of a universally superior approach.

Importantly, similar ROM outcomes across protocols suggest that rehabilitation schedules can be flexibly adapted, provided functional milestones are met. Collectively, these findings support a more patient-centered model of postoperative care one that embraces tailored rehabilitation, prioritizes opioid stewardship, and elevates patient satisfaction as a core outcome.

### Strengths and limitations

Strengths of this review included its focus on PROMs such as ASES, SST, SANE, and QoR-15, offering a multidimensional perspective on patient outcomes. It also captured emerging perioperative trends in ERAS and OS strategies, aligning with contemporary priorities in orthopedic surgery.

Limitations included methodological heterogeneity and variation in follow-up durations, which precluded meta-analysis. Inconsistent reporting of secondary outcomes (eg, SST and QoR-15) limited cross-study comparison. Several included studies lacked randomization and blinding, increasing the risk of bias. Additionally, small sample sizes in many studies reduce generalizability.

#### Future directions

Future research should prioritize level 1 high-quality, adequately powered, multicenter randomized trials to define the optimal timing of rehabilitation and clarify whether delayed mobilization confers sustained functional benefits without added risk. Comparative studies on analgesic modalities particularly continuous versus single-shot interscalene blocks and LB are required, with longer follow-up to assess durability of outcomes and patient satisfaction.

Standardizing the use of outcome measures such as QoR-15 and SANE will enhance comparability and enable meta-analytical synthesis. Large-scale registry data and qualitative studies can further illuminate patient-centered recovery, safety, and cost-effectiveness across diverse perioperative pathways. Looking ahead, personalized rehabilitation and analgesic strategies, supported by predictive analytics, hold promise for optimizing outcomes in SA.

## Conclusion

This systematic review demonstrated that diverse perioperative strategies ranging from delayed rehabilitation to opioid-free analgesia can yield comparable functional recovery following SA. Delayed mobilization may improve midterm ASES outcomes, while OS pathways offer equal or better satisfaction without compromising patient safety. CISBs and LB remain viable analgesic options, although selection should be individualized. These findings support a tailored, patient-centered approach that prioritizes functional outcomes, satisfaction, and safety, and underscore the need for further high-quality studies to refine evidence-based perioperative protocols.

## Disclaimers:

Funding: No funding was disclosed by the authors.

Conflicts of interest: The author, their immediate family, and any research foundation with which they are affiliated have not received any financial payments or other benefits from any commercial entity related to the subject of this article.
