# Supplementary material for: Impact of enhanced recovery after surgery protocols on patient-reported outcomes and satisfaction following shoulder arthroplasty: a systematic review
Source: JSES Rev Rep Tech. 2026 Mar 19;6(3):100725. doi: 10.1016/j.xrrt.2026.100725 (PMC13122315; doi:10.1016/j.xrrt.2026.100725)
Supplement: Supplementary Table 1 [file mmc1.docx]

| Search String |
| --- |
| (Enhanced Recovery After Surgery OR Fast-Track Surgery OR Enhanced Recovery Pathways OR ERAS OR Enhanced Recovery Programs OR Enhanced Recovery Protocols OR Fast Track Protocols OR Perioperative Care OR Multimodal Analgesia) AND (Shoulder Arthroplasty OR Shoulder Replacement OR Total Shoulder Arthroplasty OR Reverse Shoulder Arthroplasty OR Hemiarthroplasty OR Shoulder Prosthesis) AND (Postoperative Care OR Pain Management OR Rehabilitation OR Length of Stay OR Hospital Stay OR Complications OR Functional Outcome OR Opioid-Sparing OR Analgesia OR Physical Therapy Modalities) |

Supplementary Table 1 - Search String
