# Supplementary material for: Impact of enhanced recovery after surgery protocols on patient-reported outcomes and satisfaction following shoulder arthroplasty: a systematic review
Source: JSES Rev Rep Tech. 2026 Mar 19;6(3):100725. doi: 10.1016/j.xrrt.2026.100725 (PMC13122315; doi:10.1016/j.xrrt.2026.100725)
Supplement: Supplementary Table 2 [file mmc2.docx]

| **Criteria Type** | **Inclusion Criteria** | **Exclusion Criteria** |
| --- | --- | --- |
| Population | Studies involving adult patients (≥18 years) undergoing any type of shoulder arthroplasty (total shoulder arthroplasty, reverse total shoulder arthroplasty, hemiarthroplasty) | Studies on non-shoulder procedures (e.g., hip or knee arthroplasty); paediatric populations |
| Intervention | Studies implementing one or more components of an ERAS protocol | Studies without any identifiable ERAS components or those not evaluating perioperative protocols |
| Comparator | Studies with a comparison to standard care, historical cohorts, or pre-ERAS implementation data | Studies without a comparator group or baseline for evaluation |
| Outcomes | Studies reporting on at least one of the following outcomes: PROMs (ASES, SST), QoR-15, ROM, Satisfaction | Studies that do not report any relevant postoperative outcome data |
| Study Type | Randomised controlled trials, cohort studies (prospective or retrospective), case-control studies, and quasi-experimental designs | Case reports, editorials, narrative reviews, protocols, and expert opinions |
| Language | Studies published in English | Non-English language studies without an available translation |
| Publication Date | No restriction on date of publication | None |

Supplementary Table 2 – Inclusion and Exclusion Criteria
