# Supplementary material for: Impact of enhanced recovery after surgery protocols on patient-reported outcomes and satisfaction following shoulder arthroplasty: a systematic review
Source: JSES Rev Rep Tech. 2026 Mar 19;6(3):100725. doi: 10.1016/j.xrrt.2026.100725 (PMC13122315; doi:10.1016/j.xrrt.2026.100725)
Supplement: Supplementary Table 3 [file mmc3.docx]

| **Component** | **Description** |
| --- | --- |
| P (Population) | Adult patients undergoing shoulder arthroplasty (including total shoulder arthroplasty, reverse total shoulder arthroplasty, hemiarthroplasty) for any indication (e.g., osteoarthritis, rotator cuff disease, fractures) |
| I (Intervention) | Implementation of an Enhanced Recovery After Surgery (ERAS) protocol—defined as a multimodal, evidence-based perioperative care pathway that may include preoperative education, optimised analgesia, early mobilisation, nutritional support, and standardised anaesthesia |
| C (Comparison) | Standard or conventional perioperative care pathways without ERAS components |
| O (Outcomes) | Functional PROMs and Patient satisfaction |

Supplementary Table 3 – PICO Framework for the systematic review
